# The Association Between Serum Uric Acid Levels and the Risk of Cognitive Dysfunction in Patients With Atrial Fibrillation

**DOI:** 10.1155/ije/2221976

**Published:** 2024-12-07

**Authors:** Miaomiao Shang, Meijuan Wang, Qian Cui, Dongmei Song, Wenqing Wang, Jing Xue, Guomei Xu, Dandan Sun

**Affiliations:** Department of Cardiology, Affiliated Hospital of Jining Medical University, Jining, Shandong, China

**Keywords:** atrial fibrillation, cognitive dysfunction, nonlinear relationship, serum uric acid

## Abstract

Patients with atrial fibrillation (AF) are linked to an increased risk of cognitive dysfunction, and serum uric acid levels play an important factor in cognitive dysfunction. However, the optimal serum uric acid level in patients with AF remains unclear. Therefore, we aimed to explore the relationship between serum uric acid and cognitive dysfunction. 583 patients were conducted in the Affiliated Hospital of Jining Medical University. Cognitive dysfunction was assessed by the Montreal Cognitive Assessment (MoCA). The relationship between serum uric acid levels and the risk of cognitive dysfunction in patients with AF was analyzed using the smoothing spline fitting model and threshold analysis. The average serum uric acid level was (383.26 ± 110.11) μmol/L, and the incidence of cognitive dysfunction was 79.76%. There was a non-linear relationship between serum uric acid levels and the risk of cognitive dysfunction in patients with AF, and the inflection point was 352 μmol/L. At the left of the inflection point, the relationship was significant (OR = 1.02, 95% CI = 1.00–1.04). At the right of the inflection point, there was no statistical difference (*p*=0.101). When serum uric acid levels are less than 352 μmol/L, the risk of cognitive dysfunction increases by 2% for each unit increase in serum uric acid levels in patients with AF. The study provides evidence for the treatment of serum uric acid levels in patients with AF.

## 1. Introduction

Atrial fibrillation (AF) is the most common type of arrhythmia in adults, with an estimated more than 8 million people in China suffering from AF at 45 years old [1]. One of the pathogenesis of AF is inflammation and oxidative stress [2]. Uric acid or enzymes in the producing pathway of uric acid are associated with oxidative stress, inflammation, and endothelial dysfunction, all of which may play a role in the development and progression of AF [3, 4]. High serum uric acid levels have been reported to increase the risk of stroke in patients with AF [5], which in turn increases the risk of cognitive impairment. However, there is growing evidence that the association between AF and cognitive impairment is independent of clinical stroke [6]. Studies have shown that patients with AF had a 1.4-fold increased risk of developing cognitive dysfunction compared to the general population [7, 8]. In addition, other studies have shown that elevated serum uric acid levels lead to decreased brain metabolism, which may be significantly related to the development of cognitive dysfunction [9–11]. It is worth emphasizing that the prevalence of hyperuricemia has risen dramatically, and the current incidence of hyperuricemia in China is 13.3% [12]. At present, hyperuricemia has become the second most common metabolic disease after diabetes. However, the effect of serum uric acid levels on cognitive dysfunction remains unclear in patients with AF. Further investigation is needed to determine the accurate levels of serum uric acid in improving the cognitive function in patients with AF. Therefore, this study aims to explore the relationship between serum uric acid levels and the occurrence of cognitive dysfunction in patients with AF, so as to help clinicians make the optimal decision in controlling serum uric acid.

## 2. Methods

**Figure 1:**
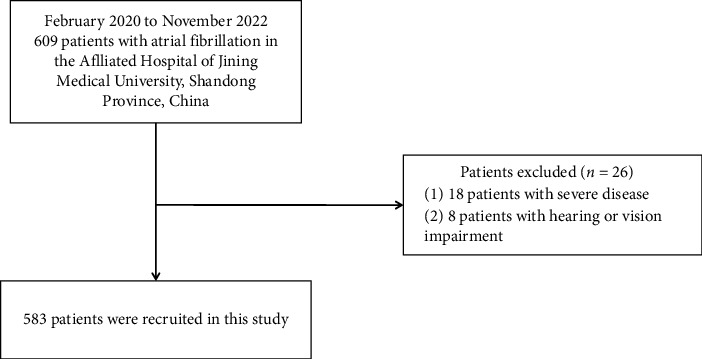
The flowchart of patients selection.

This investigation was a descriptive cross-sectional design. From February 2020 to November 2022, 583 participants of AF who were hospitalized in the Affiliated Hospital of Jining Medical University were recruited into the investigation. The inclusion criteria were as follows: (1) according to the 2019 AHA/ACC/HRS Focused Update of the 2014 AHA/ACC/HRS Guideline for the Management of Patients with AF [13], the diagnosis of AF was based on 12-lead electrocardiography or 24-h Holter monitoring; (2) all participants signed the informed consent forms. The exclusion criteria were as follows: (1) patients with severe liver and kidney function damage or malignant tumor; (2) patients with hearing or vision impairment and were unable to complete the questionnaire.  Figure 1 is a flowchart of patient selection. This study was approved by the Ethics Committee of the Affiliated Hospital of Jining Medical University (NO: 2020C009).

The following data were collected: (1) General information: including age, gender, life status, degree of education, marital status, smoking status, and alcohol consumption. (2) Clinical data: including valvular heart disease, hypertension, diabetes, heart failure, coronary heart disease, myocardial infarction, hyperlipidemia, cerebral infarction, AF, body mass index, red blood cell, hemoglobin, white blood cell, platelets, free triiodothyronine, free thyroxine, creatinine, left ventricular ejection fraction, left atrial diameter, and medicine use (aspirin, warfarin, clopidogrel, and amiodarone). All biochemistry analyses were performed in the biochemistry laboratory using standard automated procedures. Patients were asked to remove their coats and shoes during weight measurement. Echocardiography was performed by qualified sonographers, and researchers were responsible for collecting relevant indicators.

Cognitive function was measured using the MoCA-Beijing. The scale has some linguistic and cultural modifications to the original scale. The MoCA-Beijing is a 30 points scale with six cognitive subtests: (1) short-term memory; (2) visuospatial competence; (3) execution ability; (4) attention, numeracy, and working memory; (5) language; and (6) orientation. If the participant has less than 12 years of education, one point is added to the total score. Higher scores indicate better cognitive function. When the score is less than 26 points, the patient is recognized as having cognitive dysfunction. MoCA was performed face-to-face by the investigator in strict accordance with the guidelines, and it took about 10–15 min to complete. The MoCA had a high sensitivity of 0.85 [14].

K-S method was used to test the normality of continuous variables. Variables with normal distribution were represented by *x* ± *s* and one-way ANOVA analysis was used. Variables with non-normal distribution were represented by median (minimum, maximum), and the Kruskal–Wallis H test was used. *N* (%) and the Chi square test were used for categorical variables. First, univariate analysis was performed to compare baseline characteristics in patients with AF with different serum uric acid levels. Secondly, univariate analysis was used to analyze the occurrence of cognitive dysfunction. At last, three models were constructed in this study to explore the relationship between serum uric acid level and cognitive dysfunction in different models. Model 1, Model 2, and Model 3 represent rough models without adjusting any covariates, adjusted only for sociodemographic variables and adjusted the model for all covariates, respectively. The threshold effect test and smoothing spline fitting were used to manage the nonlinear relationship between the occurrence of serum uric acid and cognitive dysfunction. *p* < 0.05 was considered to be statistically significant. All analyses were conducted using SPSS 22.0 and EmpowerStats 4.1 software.

## 3. Results

In this study, 583 patients with AF were collected, with an average age of 66.96 ± 10.62 years. Of these, 333 were men, accounting for 57.12%. The average serum uric acid level was (383.26 ± 110.11) μmol/L, and the incidence of cognitive dysfunction was 79.76%. At present, the definition of hyperuricemia is not uniform. Considering that the lifelong risk of gout is significantly increased when the serum uric acid level exceeds 360 μmol/L (6 mg/dL), 360 μmol/L has been proposed as the threshold for defining hyperuricemia [15, 16]. In addition, when the serum uric acid concentration exceeds 420 μmol/L, monosodium urate crystals can directly adhere to and deposit in joints, surrounding soft tissues, kidney tissues, and blood vessels. This deposition can lead to damage in multiple organs, including the heart, brain, and kidneys [17, 18]. So some experts define hyperuricemia as a serum uric acid level over 420 μmol/L (7 mg/dL) [12]. Therefore, the patients were divided into three groups: T1 (≤ 360), T2 (> 360, ≤ 420), and T3 (> 420). There were significant differences in gender, live status, marital status, diabetes, heart failure, coronary heart disease, myocardial infarction, medicine use (aspirin, amiodarone), hemoglobin, platelets, free triiodothyronine, creatinine, and left ventricular ejection fraction among the three groups (*p* < 0.05).  Table 1 presents the baseline characteristics.

Univariate analysis showed that age (OR = 1.09, 95% CI: 1.07–1.12), gender (OR = 4.20, 95% CI: 2.55, 6.91), primary school (OR = 0.06, 95% CI: 0.02, 0.19), high school and above (OR = 0.01, 95% CI: 0.00, 0.03), hyperlipidemia (OR = 0.20, 95% CI: 0.09, 0.45), red blood cell (OR = 0.67, 95% CI: 0.49, 0.93), hemoglobin (OR = 0.98, 95% CI: 0.97, 0.99), free thyroxine (OR = 1.12, 95% CI: 1.06, 1.18) and body mass index (OR = 0.93, 95% CI: 0.89, 0.97) were significantly related to the occurrence of cognitive dysfunction(*p* < 0.05) (Table 2).

In this study, three models were constructed to explore the effect of serum uric acid levels on cognitive dysfunction. In Model 3 which we adjusted for all variables, the effect size of serum uric acid was 1.01. For each unit increase in serum uric acid levels, the risk of cognitive dysfunction increased by 1% (95% CI: 1.00–1.02, *p*=0.117). The risk of cognitive dysfunction in the T2 group was 4.01 times that of the T1 group, and the risk of cognitive dysfunction in the T3 group was 2.32 times that of the T1 group. In the sensitivity analysis, when serum uric acid levels were transformed into a categorical variable, the *p* value of the trend of the risk of cognitive dysfunction with serum uric acid was consistent with that when serum uric acid was analyzed as a continuous variable (Table 3).

**Figure 2:**
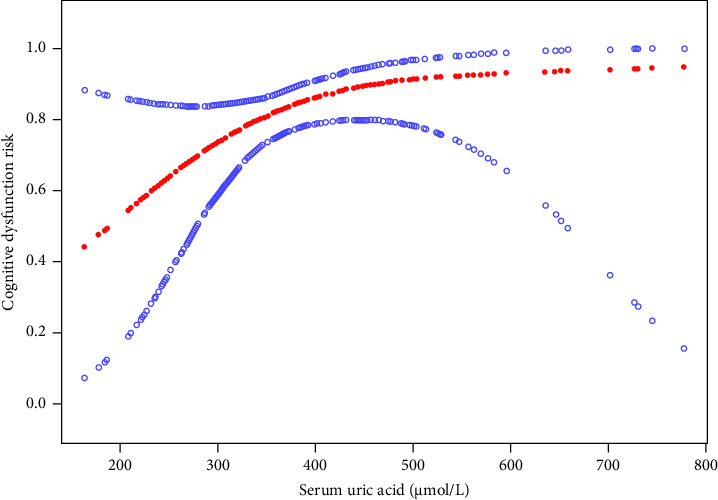
Association between serum uric acid and cognitive dysfunction risk of atrial fibrillation. A threshold, nonlinear relationship between serum uric acid and cognitive dysfunction risk was found in a generalized additive model. The solid red line represents the smooth curve fit between variables. Blue bands represent the 95% confidence interval from the fit.

Smooth spline fitting and threshold effect test results showed that serum uric acid levels were curve-related to the risk of cognitive dysfunction. Using multiple regression and recursion algorithms, the inflection point is calculated at 352 μmol/L. On the left side of the inflection point, the relationship was significant (OR = 1.02, 95% CI = 1.00–1.04). On the right side of the inflection point, there was no significant (*p*=0.101) (Table 4, Figure 2).

## 4. Discussion

The results of this study showed that there were differences in serum uric acid levels among people with different gender, living status, marital status, diabetes, coronary heart disease, myocardial infarction, heart failure, left ventricular ejection fraction, hemoglobin, creatinine, platelet, left atrial diameter, and medicine use (aspirin and amiodarone). Among the patients in the T3 group, the proportion of patients living alone was relatively high, which may be related to the patient's poor lifestyle and dietary patterns such as smoking, drinking, high-purine diet, and high-fat diet. In general, patients with a history of heart failure have a lower left ventricular ejection fraction and an increased left atrial diameter. In addition, myocardial infarction, heart failure, and elevated serum uric acid are associated with some common risk factors. For example, dyslipidemia, obesity, frequent consumption of fried foods, smoking, and alcohol consumption [19–24]. Serum uric acid also interacts with multiple serum markers (creatinine, hemoglobin, and platelets) as a dynamic oxidative stress indicator. Researches demonstrate that hyperuricemia is associated with type 2 diabetes. Serum uric acid induces insulin resistance and gluconeogenesis by inhibiting AMP-activated protein kinase, and insulin resistance also reduces urate excretion [25, 26]. Sex hormones also affect the excretion of uric acid, which is an important reason for the difference in serum uric acid in patients of different genders [27]. Studies have shown that patients with coronary heart disease have increased serum uric acid levels compared with patients without coronary heart disease, and the incidence of hyperuricemia in patients with coronary heart disease is greatly increased, which is considered to be an indicator of cardiovascular disease prognosis [28]. Aspirin can inhibit the aggregation of platelets, improve glomerular circulation, and excrete more uric acid [29]. As a result, patients taking aspirin had lower serum uric acid levels. Different marital status and amiodarone use may be affected by sample size. In general, individualized treatment is required when adjusting the patient's serum uric acid level.

Previous studies have shown that age, degree of education, heart failure, hypotension, diabetes, chronic kidney disease, and alcohol consumption are risk factors for cognitive dysfunction in patients with AF [30–32]. Results of this study showed that age, gender, degree of education, aspirin use for more than 1 month, body mass index, free thyroxine, and other indicators were significantly associated with the occurrence of cognitive dysfunction in patients with AF. However, the mechanism of cognitive dysfunction in patients with AF is not fully understood. The relevant possible mechanisms include the following [33]: (1) hypercoagulable state of blood; (2) the formation of inflammatory cytokine; (3) cerebral hypoperfusion; and (4) genes and other factors. Diener [30] also pointed out that further research and exploration of the mechanism of cognitive dysfunction in patients with AF is needed.

An independent association between elevated serum uric acid levels and diabetes is evident in some studies [34]. Diabetes is not only one of the risk factors for AF, but is also related to cognitive function [35, 36]. In addition, a positive correlation between education level and cognitive function has been consistently demonstrated [35]. Therefore, this study still showed a correlation between serum uric acid levels and cognitive impairment in patients with AF after controlling for relevant influencing factors. The inflection point of the relationship is 352 μmol/L, and there is no significant correlation on the right side of the inflection point. On the left side of the inflection point, the risk of cognitive dysfunction increases by 2% for each unit increase in serum uric acid levels in patients with AF. Previous studies have shown that high serum uric acid levels are an independent risk factor for the development of vascular dementia [37, 38], which is consistent with the results of our study. The mechanisms of cognitive dysfunction caused by elevated serum uric acid levels include the following four aspects: (1) uric acid has the effect of promoting inflammation and oxidative stress, which can lead to vascular endothelial dysfunction and directly promote the formation of atherosclerosis [39]; (2) elevated uric acid will increase the burden of cerebral ischemia, resulting in demyelinating changes in brain tissue and increasing brain protein hypersignal; (3) when serum uric acid levels increase, urate is deposited in the vessel wall. That leads to endothelial injury and interstitial fluid leakage, which promotes dilation of the perivascular space [10, 40]; and (4) high serum uric acid levels lead to an imbalance of intestinal flora, which may be closely related to the development of cognitive dysfunction [41, 42]. Although serum uric acid levels of less than 360 μmol/L are in the normal range, the relevant guidelines also recommend that uric acid levels be controlled at a lower level, especially in patients with comorbidities. The serum uric acid level should be controlled below 300 μmol/L as much as possible, and it is not recommended to be lower than 180 μmol/L [43–45]. For groups with uric acid levels less than 360 μmol/L, although it is not necessary to use drugs, uric acid levels can be controlled through lifestyle changes. In terms of diet, the consumption of vegetables, eggs, and milk is encouraged. Limit beef, lamb, pork, and purine-rich seafood. Avoid fructose-containing beverages, animal-offal, liquor, beer, and rice wine [46]. In addition to restricting a high-purine diet, smoking cessation, weight control (body mass index: 18.5–23.9 kg/m^2^), drinking more water (normal heart and kidney function to maintain daily urine output of 2000–3000 mL), moderate exercise (at least 150 min of moderate-intensity aerobic exercise per week) can also reduce serum uric acid levels [47–49]. Based on the results of our study, clinicians should take effective measures to control serum uric acid levels even if the serum uric acid level of patients is less than 352 μmol/L. We recommend patients improve their lifestyle and diet to reduce the risk of cognitive dysfunction.

This study explored the nonlinear correlation between serum uric acid levels and cognitive dysfunction in patients with AF. It is of great significance to establish a risk prediction model for cognitive dysfunction in patients with AF. However, this study also has some limitations. On the one hand, the number of patients with serum uric acid levels between 360 and 420 μmol/L was relatively small, possibly due to selection bias in this single-center study. This can be further verified by expanding the study's scope and increasing the sample size. On the other hand, the cross-sectional design has limitations in terms of causal inference. In addition, the use of statins and uric-lowering drugs can affect the level of serum uric acid [50]. However, information on the use of statins and uric-lowering drugs was not included in this study, which is the shortcoming of this study. We may do some in‐depth research about serum uric acid levels and cognitive function by improving research designs in the future.

## 5. Conclusions

Results of this study showed that when serum uric acid levels of patients with AF were lower than 352 μmol/L, the risk of cognitive dysfunction increased by 2% for each unit increase. However, when serum uric acid levels are more than 352 μmol/L, the relationship is not significant. This suggests that clinicians should take effective measures to control serum uric acid levels. We encourage patients to modify their lifestyle, dietary habits, and take antiacid medication in order to reduce the risk of cognitive dysfunction.

## Figures and Tables

**Table 1:** Participants characteristics by serum uric acid (*N* = 583).

Characteristic	≤ 360	> 360, ≤ 420	> 420	*p*
*N*	277 (47.51)	97 (16.64)	209 (35.85)	
Age, year	67.99 ± 10.41	66.63 ± 9.39	65.75 ± 11.31	0.267
Gender, *n* (%)				< 0.001
Male	137 (49.46)	67 (69.07)	129 (61.72)	
Female	140 (50.54)	30 (30.93)	80 (38.28)	
Degree of education, *n* (%)				0.101
Illiterate	100 (36.10)	29 (29.90)	61 (29.19)	
Primary school	125 (45.13)	41 (42.27)	111 (53.11)	
Junior school	30 (10.83)	20 (20.62)	23 (11.00)	
High school and above	22 (7.94)	7 (7.22)	14 (6.70)	
Marital status, *n* (%)				0.015
Unmarried	2 (0.72)	0 (0.00)	0 (0.00)	
Married	248 (89.53)	82 (84.54)	177 (84.69)	
Divorce	0 (0.00)	2 (2.06)	0 (0.00)	
Widowed	27 (9.75)	13 (13.40)	32 (15.31)	
Smoke, *n* (%)				0.454
Nonsmoker	170 (61.37)	53 (54.64)	121 (57.89)	
Current smoker	51 (18.41)	26 (26.80)	49 (23.44)	
Quit	56 (20.22)	18 (18.56)	39 (18.66)	
Alcohol consumption, *n* (%)				0.051
Nondrinker	211 (76.17)	61 (62.89)	144 (68.90)	
Current drinker	46 (16.61)	26 (26.80)	39 (18.66)	
Quit	20 (7.22)	10 (10.31)	26 (12.44)	
Valvular heart disease, *n* (%)				0.126
No	192 (69.31)	68 (70.10)	128 (61.24)	
Yes	85 (30.69)	29 (29.90)	81 (38.76)	
Hypertension, *n* (%)				0.176
No	136 (49.10)	37 (38.14)	96 (45.93)	
Yes	141 (50.90)	60 (61.86)	113 (54.07)	
Diabetes, *n* (%)				0.008
No	218 (78.70)	61 (62.89)	158 (75.60)	
Yes	59 (21.30)	36 (37.11)	51 (24.40)	
Heart failure, *n* (%)				< 0.001
No	144 (51.99)	32 (32.99)	65 (31.10)	
Yes	133 (48.01)	65 (67.01)	144 (68.90)	
Coronary heart disease, *n* (%)				< 0.001
No	48 (17.33)	15 (15.46)	64 (30.62)	
Yes	229 (82.67)	82 (84.54)	145 (69.38)	
Myocardial infarction, *n* (%)				0.004
No	246 (88.81)	91 (93.81)	202 (96.65)	
Yes	31 (11.19)	6 (6.19)	7 (3.35)	
Hyperlipidemia, *n* (%)				0.567
No	267 (96.39)	94 (96.91)	198 (94.74)	
Yes	10 (3.61)	3 (3.09)	11 (5.26)	
Cerebral infarction, *n* (%)				0.537
No	237 (85.56)	81 (83.51)	171 (81.82)	
Yes	40 (14.44)	16 (16.49)	38 (18.18)	
Aspirin, *n* (%)				0.036
No	171 (61.73%)	58 (59.79)	150 (71.77)	
Yes	106 (38.27%)	39 (40.21)	59 (28.23)	
Warfarin, *n* (%)				0.166
No	239 (86.28)	83 (85.57)	191 (91.39)	
Yes	38 (13.72)	14 (14.43)	18 (8.61)	
Clopidogrel, *n* (%)				0.937
No	257 (92.78)	89 (91.75)	194 (92.82)	
Yes	20 (7.22)	8 (8.25)	15 (7.18)	
Amiodarone, *n* (%)				0.050
No	269 (97.11)	95 (97.94)	209 (100.00)	
Yes	8 (2.89)	2 (2.06)	0 (0.00)	
Type of atrial fibrillation, *n* (%)				0.066
Paroxysmal	87 (31.41)	29 (29.90)	48 (22.97)	
Persistent	126 (45.49)	53 (54.64)	116 (55.50)	
Long-standing persistent	32 (11.55)	3 (3.09)	18 (8.61)	
Permanent	32 (11.55)	12 (12.37)	27 (12.92)	
Body mass index (kg/m^2^)	24.87 ± 3.48	26.27 ± 4.22	25.66 ± 5.14	0.005
Red blood cell (10^12^/L)	4.42 ± 0.55	4.56 ± 0.73	4.32 ± 0.72	0.054
Hemoglobin (g/L)	135.65 ± 16.53	140.72 ± 20.15	132.07 ± 23.85	0.016
White blood cell (10^9^/L)	7.11 ± 3.37	7.29 ± 2.68	7.03 ± 2.36	0.480
Platelets (10^9^/L)	219.07 ± 67.74	191.80 ± 61.36	196.88 ± 66.39	<0.001
Free triiodothyronine (pmol/L)	4.33 (2.13, 31.80)	4.17 (1.95, 7.04)	4.11 ± 0.99	<0.001
Free thyroxine (pmol/L)	18.70 ± 9.12	18.20 (3.72, 100.00)	18.22 ± 3.16	0.203
Creatinine (umol/L)	64.48 ± 18.22	74.56 ± 24.86	85.29 ± 23.95	<0.001
Left ventricular ejection fraction (%)	53.19 ± 9.48	46.52 ± 13.85	47.18 ± 13.12	<0.001
Left atrial diameter (mm)	45.48 ± 9.17	49.45 ± 7.92	49.29 ± 10.22	<0.001
MoCA	19.06 ± 7.01	20.25 ± 6.14	19.37 ± 6.84	0.388

**Table 2:** Univariate analysis for the risk of cognitive dysfunction.

Covariate	Statistics	OR (95% CI)	*p*
Age, year	66.96 ± 10.62	1.09 (1.07, 1.12)	< 0.001
Gender, *n* (%)			< 0.001
Male	333 (57.12)	1.0	
Female	250 (42.88)	4.20 (2.55, 6.91)	
Marital status, *n* (%)			
Unmarried	2 (0.34)	1.0	
Married	507 (86.96)	0.00 (0.00, Inf)	0.989
Divorce	2 (0.34)	1.00 (0.00, Inf)	1.000
Widowed	72 (12.35)	0.00 (0.00, Inf)	0.989
Degree of education, *n* (%)			
Illiterate	190 (32.59)	1.0	
Primary school	277 (47.51)	0.06 (0.02, 0.19)	< 0.001
Junior school	73 (12.52)	0.03 (0.01, 0.09)	< 0.001
High school and above	43 (7.38)	0.01 (0.00, 0.03)	< 0.001
Smoke, *n* (%)			
Nonsmoker	344 (59.01)	1.0	
Current smoker	126 (21.61)	0.42 (0.25, 0.69) 0.0006	< 0.001
Quit	113 (19.38)	0.32 (0.19, 0.53) < 0.0001	< 0.001
Alcohol consumption, *n* (%)			
Nondrinker	416 (71.36)	1.0	
Current drinker	111 (19.04)	0.37 (0.23, 0.59)	< 0.001
Quit	56 (9.61)	0.64 (0.32, 1.24)	0.186
Valvular heart disease, *n* (%)			0.158
No	388 (66.55)	1.0	
Yes	195 (33.45)	1.38 (0.88, 2.15)	
Hypertension, *n* (%)			0.251
No	269 (46.14)	1.0	
Yes	314 (53.86)	1.27 (0.85, 1.90)	
Diabetes, *n* (%)			0.412
No	437 (74.96)	1.0	
Yes	146 (25.04)	0.83 (0.52, 1.30)	
Heart failure, *n* (%)			0.275
No	241 (41.34)	1.0	
Yes	342 (58.66)	1.25 (0.84, 1.88)	
Coronary heart disease, *n* (%)			0.187
No	127 (21.78)	1.0	
Yes	456 (78.22)	1.37 (0.86, 2.19)	
Myocardial infarction, *n* (%)			0.458
No	539 (92.45)	1.0	
Yes	44 (7.55)	1.37 (0.60, 3.16)	
Hyperlipidemia, *n* (%)			< 0.001
No	559 (95.88)	1.0	
Yes	24 (4.12)	0.20 (0.09, 0.45)	
Cerebral infarction, *n* (%)			0.397
No	489 (83.88)	1.0	
Yes	94 (16.12)	1.28 (0.72, 2.30)	
Aspirin, *n* (%)			0.021
No	379 (65.01)	1.0	
Yes	204 (34.99)	0.62 (0.41, 0.93)	
Warfarin, *n* (%)			0.127
No	513 (87.99)	1.0	
Yes	70 (12.01)	0.64 (0.36, 1.14)	
Clopidogrel, *n* (%)			0.073
No	540 (92.62)	1.0	
Yes	43 (7.38)	2.61 (0.91, 7.45)	
Amiodarone, *n* (%)			0.131
No	573 (98.28)	1.0	
Yes	10 (1.72)	0.37 (0.10, 1.34)	
Type of atrial fibrillation, *n* (%)			
Paroxysmal	164 (28.13)	1.0	
Persistent	295 (50.60)	1.06 (0.66, 1.70)	0.799
Long-standing persistent	53 (9.09)	1.17 (0.53, 2.55)	0.699
Permanent	71 (12.18)	1.21 (0.60, 2.46)	0.596
Red blood cell (10^12^/L)	4.41 ± 0.65	0.67 (0.49, 0.93)	0.016
Hemoglobin (g/L)	135.22 ± 20.21	0.98 (0.97, 0.99)	< 0.001
White blood cell (10^9^/L)	7.11 ± 2.93	1.09 (1.00, 1.18)	0.055
Platelets (10^9^/L)	206.54 ± 67.20	1.00 (1.00, 1.00)	0.443
Free triiodothyronine (pmol/L)	4.86 ± 3.30	1.07 (0.98, 1.18)	0.151
Free thyroxine (pmol/L)	18.78 ± 9.42	1.12 (1.06, 1.18)	< 0.001
Creatinine (umol/L)	73.56 ± 23.51	1.00 (0.99, 1.01)	0.722
Body mass index (kg/m^2^)	25.39 ± 4.29	0.93 (0.89, 0.97)	0.001
Left ventricular ejection fraction (%)	49.85 ± 12.10	1.00 (0.98, 1.01)	0.700
Left atrial diameter (mm)	47.56 ± 9.56	0.97 (0.95, 1.00)	0.018

**Table 3:** Effect of serum uric acid levels on the risk of cognitive dysfunction.

Model 1			Model 2		Model 3	
Variable	OR (95% CI)	*p*	OR (95% CI)	*p*	OR (95% CI)	*p*
UA	1.00 (1.00, 1.00)	0.753	1.00 (1.00, 1.00)	0.053	1.01 (1.00, 1.02)	0.002
UA						
T1	Reference		Reference		Reference	
T2	1.27 (0.71, 2.27)	0.429	1.86 (0.97, 3.55)	0.061	4.01 (0.71, 22.74)	< 0.001
T3	1.30 (0.83, 2.04)	0.257	2.20 (1.30, 3.73)	0.003	2.32 (1.43, 4.11)	< 0.001
*p* for trend	1.14 (0.91, 1.43)	0.244	1.50 (1.15, 1.95)	0.002	3.15 (2.35, 5.44)	< 0.001

*Note:* Model 1: adjusted for none. Model 2: adjusted for age, gender. Model 3: adjusted for age, gender, degree of education, smoke, alcohol consumption, valvular heart disease, hypertension, diabetes, heart failure, coronary heart disease, myocardial infarction, hyperlipidemia, cerebral infarction, aspirin, warfarin, clopidogrel, amiodarone, type of atrial fibrillation, red blood cell, hemoglobin, free thyroxine, BMI, left atrial diameter.

**Table 4:** Threshold analysis of serum uric acid levels on the risk of cognitive dysfunction.

Threshold (μmol/L)	Point of effect size	95% CI	*p*
< 352	1.02	(1.00, 1.04)	0.030
≥ 352	0.98	(0.95, 1.00)	0.101

## Data Availability

The datasets collected and analyzed in this study are available from the corresponding author upon reasonable request.
